# Pulmonary Embolism as the Initial Presentation of Testicular Carcinoma

**DOI:** 10.1155/2013/264569

**Published:** 2013-12-09

**Authors:** Ilhami Berber, Recep Bentli, Mehmet Ali Erkurt, Ozkan Ulutas, Caner Ediz, Ilknur Nizam, Nurcan Kırıcı Berber, Serkan Unlu, Reyhan Koroglu, Mustafa Koroglu, Nusret Akpolat

**Affiliations:** ^1^Department of Hematology, Faculty of Medicine, Inonu University, 44280 Malatya, Turkey; ^2^Internal Medicine, Faculty of Medicine, Inonu University, 44280 Malatya, Turkey; ^3^Department of Nephrology, Faculty of Medicine, Inonu University, 44280 Malatya, Turkey; ^4^Department of Urology, Faculty of Medicine, Inonu University, 44280 Malatya, Turkey; ^5^Department of Chest, Faculty of Medicine, Inonu University, 44280 Malatya, Turkey; ^6^Department of Radiology, Faculty of Medicine, Inonu University, 44280 Malatya, Turkey; ^7^Department of Nuclear, Faculty of Medicine, Inonu University, 44280 Malatya, Turkey; ^8^Department of Pathology, Faculty of Medicine, Inonu University, 44280 Malatya, Turkey

## Abstract

*Objective*. The risk of pulmonary embolism is well recognized as showing an increase in oncological patients. We report a case presenting with pulmonary embolism initially, which was then diagnosed with testicular cancer. *Clinical Presentation and Intervention*. A 25-year-old man was admitted to the emergency department with a complaint of dyspnoea. Thoracic tomography, lung ventilation/perfusion scintigraphy, and an increased D-dimer level revealed pulmonary embolism. For the aetiology of pulmonary embolism, a left orchiectomy was performed and the patient was diagnosed with a germinal cell tumour of the testicle. *Conclusion*. In this paper, we present a patient for whom pulmonary embolism was the initial presentation, and a germinal cell tumour was diagnosed later during the search for the aetiology.

## 1. Introduction

Pulmonary embolism (PE) is responsible for approximately 100,000 to 200,000 deaths in the United States each year. This condition may have several clinical presentations, with a diverse range, from asymptomatic to death. The lower extremities or pelvic veins appear to be common origins of PE. There may be various risk factors for embolism, and Virchow's triad (hypercoagulability, venous stasis, and vessel wall injury) provides a model for understanding most of them. These risk factors are usually divided into two groups: inherited or acquired, where acquired reasons mostly begin with recent immobilization (myocardial infarction, surgery, recent trauma, advanced age, malignancy, and indwelling venous catheter) [1]. The risk of venous thromboembolism is now recognized to be increased in oncological patients. The annual incidence of a first episode of deep vein thrombosis or PE in the general population is 117 in 100,000. Cancer alone was associated with a 4.1-fold risk for thrombosis [2]. Otten et al. reported a study in 2004 that revealed that the overall risk for venous thrombosis increases 7-fold in patients with malignancies [3].

To the best of our knowledge, this is the first reported case that presented with PE initially, and was then diagnosed with a testicular cancer.

## 2. Case Report

A 25-year-old, previously healthy, male smoker was admitted to the emergency department of Inonu University Hospital, with complaints of sudden onset pain in the right side of his chest and dyspnoea. Upon admission, he was well oriented but appeared mildly distressed. His body temperature was 36°C, pulse rate was 120 bpm, and respiratory rate was 26/min. The chest auscultation was normal upon physical examination. No abnormally enlarged lymph nodes were palpable on any part of his body, and the abdomen was not distended. The spleen and the liver were not palpable.

The laboratory values were as follows: leukocyte count 9.100/*μ*L, haemoglobin 13.6 g/dL, haematocrit 39.5%, platelet count 152,000/*μ*L, prothrombin time 10 seconds, partial thromboplastin time 30 seconds, lactate dehydrogenase 125 U/L, total bilirubin 0.2 mg/dL, indirect bilirubin 0.1 mg/dL, and D-dimer 1.50 microgram (normal range: 0–0.5). A peripheral blood smear revealed 75% neutrophils, 20% lymphocytes, and 5% monocytes, where platelets formed clusters and the red blood cell morphology was normal. Serological examinations for human immunodeficiency virus and hepatitis B and C were all negative.

**Figure 1:**
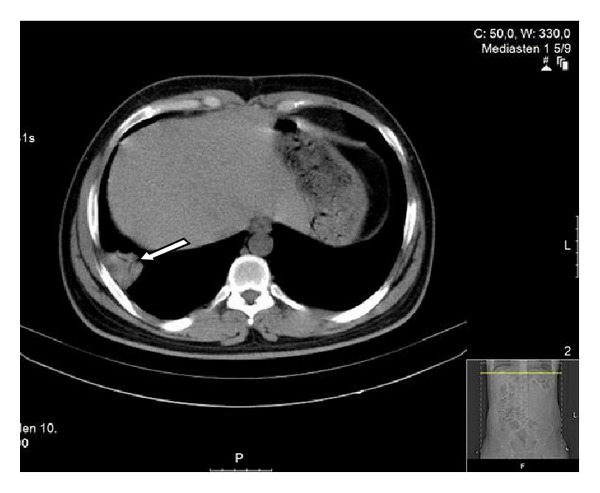
Wedge-like consolidated area.

**Figure 2:**
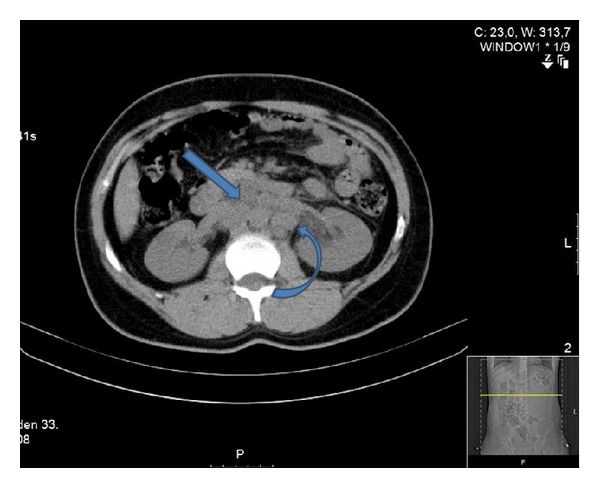
Para-aortic extensive lymphadenopathy in abdominal CT.

**Figure 3:**
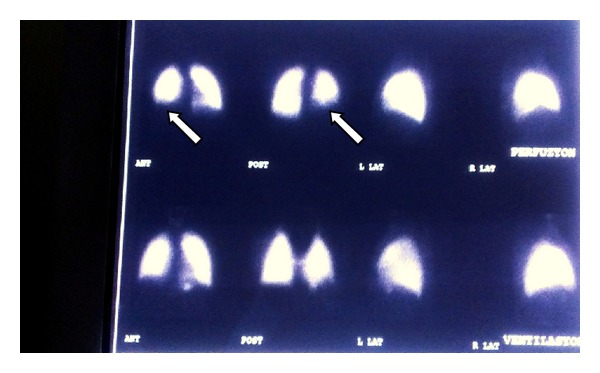
Decreased perfusion in the lower lobe of the right lung.

The patient's mother was allergic to contrast agent, therefore the patient refused to receive any contrast agent. The plain chest radiography was normal; however, the thoracic computed tomography (CT) showed a pleural-based wedge-like consolidated area and air bronchograms in the lateral posterior basal segment of the right lower lung (infarction? pneumonia?) (Figure 1). The patient's abdominal ultrasonography was normal; however, the abdominal CT showed para-aortic extensive lymphadenopathy (Figure 2). With lung ventilation/perfusion scintigraphy, there was an incompatible ventilation/perfusion ratio in the lower lobe of the right lung, assessed as high risk of pulmonary embolism (Figure 3). Doppler ultrasonography of the right and left legs was normal.

The patient was diagnosed with PE and started on a therapeutic dose of warfarin at 5 mg/day/PO, with enoxaparin sodium 2 × 60 mg/day/subcutaneous for three days. The enoxaparin sodium was stopped after 3 days, but warfarin treatment was continued. The patient's dyspnoea and pain improved on the 3rd day of treatment. For the aetiology of PE, malignant diseases were sought in addition to coagulation deficiencies. The tumour markers for testicular malignancies were evaluated, showing a *β*-human chorionic gonadotropin of 864 mIU/mL (0–25 mLU/mL) and *∂*-fetoprotein of 101 IU/mL (0.5–5.5 IU/mL). Although there was no formal histological confirmation, the tumour markers, clinical findings, and abdominal computed tomography (CT) strongly suggested the diagnosis of a metastatic testicular tumour.

**Figure 4:**
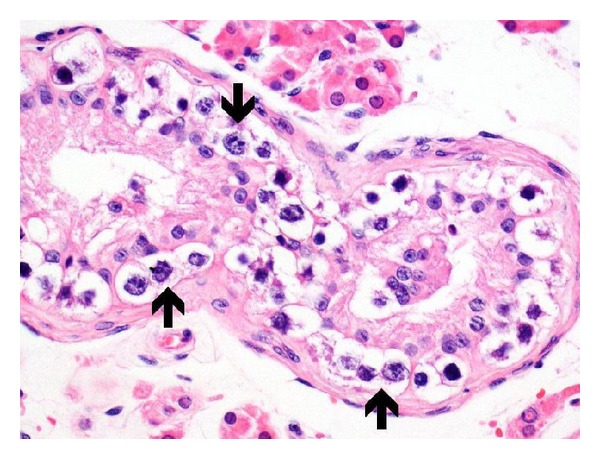
A large number of germ cells (arrows) in dysplastic tubules. A clear halo is seen around them (H&E, 400x).

**Figure 5:**
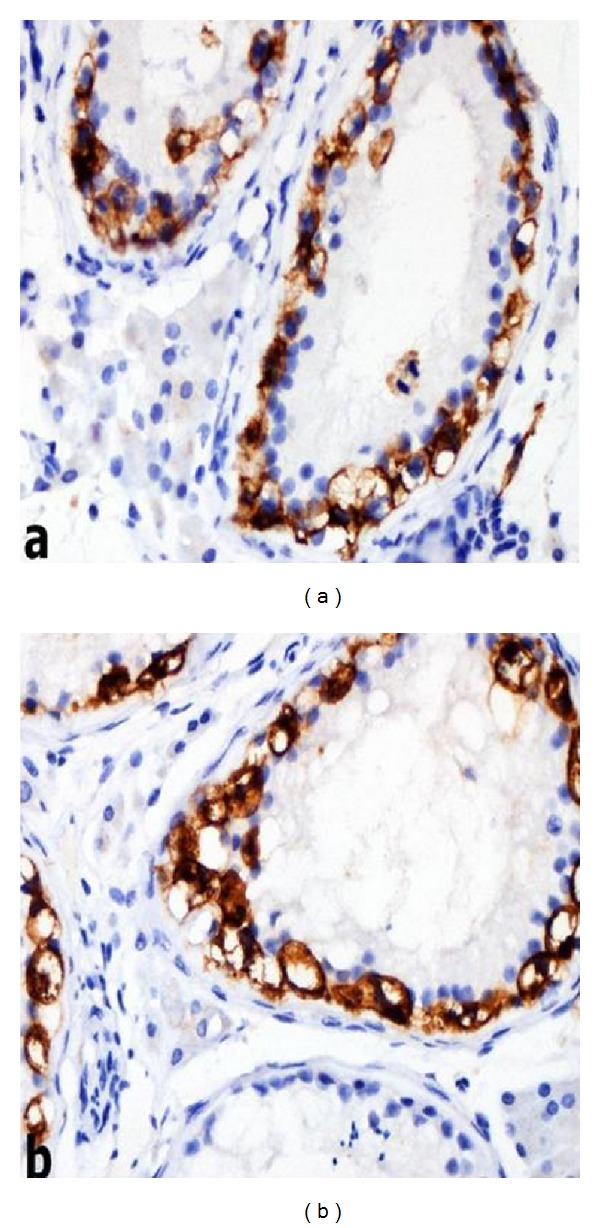
Intratubular neoplastic germ cells show up positive with antibodies of CD117 (a) and PLAP (b) in immunohistochemical staining (immunoperoxidase; 400x).

The patient was consulted with the urology department, and a testicular mass in the left testis was detected upon physical examination. Scrotal Doppler ultrasonography revealed a 5 × 2 by 5 × 7 mm-sized calcified focus with no acoustic shadowing. A left orchiectomy was performed and pathological investigation revealed a malignant germinal cell tumour of the testicle (CD117 (diffuse +), placental/germ cell alkaline phosphatase (diffuse +), and periodic acid Schiff (+)) (Figures 4, 5(a), and 5(b)).

## 3. Discussion

Tumour cells can activate blood coagulation through the production of procoagulant, fibrinolytic and proaggregating mediators, the release of proangiogenic and proinflammatory cytokines, and direct interaction with host vascular and blood cells (endothelial cells, leukocytes, and platelets) [4]. Previous studies have shown that oncological patients develop venous thromboemboli significantly more frequently and that venous thromboemboli have a significantly worse prognosis in these patients than that in nononcological patients. Trujillo-Santos et al. reported a study including 2474 patients with malignancies and acute venous thromboemboli and showed that PE was the second most frequent cause of death [5]. Bach et al. reported a new study about PE in oncological patients in 2013. They divided the PE patients into two groups: suspected (symptomatic) PE and unsuspected PE (including clinically unsuspected events that were identified in the CT examination). PE was found in 240 of 3,270 oncological patients. Of these 240 patients, 111 patients were symptomatic PE and the others were unsuspected.

In this study, 118 of 3,270 total patients had testicular cancer, and 31 of the testicular cancer patients had metastases. Only one of 240 patients had testicular cancer and suspected PE. The risk of developing PE was 1.5 times higher in patients with metastases compared to patients without metastases. Age and sex had no influence on PE risk and embolus burden [6]. Our patient was a 25-year-old male smoker. Smoking can trigger malignancy and venous thromboembolism, and he was diagnosed with testicular cancer and metastases.

Testicular cancer is a relatively rare cancer that accounts for about 1–1.5% of male cancers and mainly affects younger men in their third or fourth decade of life [7]. It can be classified into three categories: germ cell tumours (90–95%), cord stromal tumours, and miscellaneous germ cell/sex cord stromal tumours [8]. It normally appears as a painless, unilateral mass in the scrotum, or the casual discovery of an intrascrotal mass [9]. Only 1-2% of cases are bilateral at diagnosis. Cases can rarely present with pulmonary and mediastinal metastatic disease and its complications [10]. Our case did not describe testicular pain but was admitted to the emergency service with sudden onset pain in the right side and dyspnoea. He was diagnosed with PE and testicular cancer.

Physicians should consider the possibility of testicular cancer when a young male patient is diagnosed with pulmonary embolism, as in this case.
